# Transplantation of Hearts Donated after Circulatory Death

**DOI:** 10.3389/fcvm.2018.00008

**Published:** 2018-02-13

**Authors:** Christopher W. White, Simon J. Messer, Stephen R. Large, Jennifer Conway, Daniel H. Kim, Demetrios J. Kutsogiannis, Jayan Nagendran, Darren H. Freed

**Affiliations:** ^1^Cardiac Surgery, University of Alberta, Edmonton, AB, Canada; ^2^Papworth Hospital NHS Foundation Trust, Cambridge, United Kingdom; ^3^Cardiology, University of Alberta, Edmonton, AB, Canada; ^4^Critical Care Medicine, University of Alberta, Edmonton, AB, Canada; ^5^Department of Physiology, University of Alberta, Edmonton, AB, Canada; ^6^Department of Biomedical Engineering, University of Alberta, Edmonton, AB, Canada

**Keywords:** donation after circulatory death heart transplantation, donation after circulatory death cardiac graft, *Ex vivo* heart perfusion, *ex situ* heart perfusion, ischemic post-conditioning

## Abstract

Cardiac transplantation has become limited by a critical shortage of suitable organs from brain-dead donors. Reports describing the successful clinical transplantation of hearts donated after circulatory death (DCD) have recently emerged. Hearts from DCD donors suffer significant ischemic injury prior to organ procurement; therefore, the traditional approach to the transplantation of hearts from brain-dead donors is not applicable to the DCD context. Advances in our understanding of ischemic post-conditioning have facilitated the development of DCD heart resuscitation strategies that can be used to minimize ischemia-reperfusion injury at the time of organ procurement. The availability of a clinically approved *ex situ* heart perfusion device now allows DCD heart preservation in a normothermic beating state and minimizes exposure to incremental cold ischemia. This technology also facilitates assessments of organ viability to be undertaken prior to transplantation, thereby minimizing the risk of primary graft dysfunction. The application of a tailored approach to DCD heart transplantation that focuses on organ resuscitation at the time of procurement, *ex situ* preservation, and pre-transplant assessments of organ viability has facilitated the successful clinical application of DCD heart transplantation. The transplantation of hearts from DCD donors is now a clinical reality. Investigating ways to optimize the resuscitation, preservation, evaluation, and long-term outcomes is vital to ensure a broader application of DCD heart transplantation in the future.

Cardiac transplantation is the “gold-standard” treatment for eligible patients with advanced heart failure. Despite adverse changes in the donor and recipient populations, posttransplant outcomes continue to improve with a median survival of 11 years (1). While the number of potentially eligible transplant recipients is increasing, the number of transplants performed each year in Canada has remained static (2). Over the last 10 years the annual mortality rate for patients awaiting cardiac transplantation was 16% (3, 4). Overall, the clinical impact of cardiac transplantation is limited by a critical shortage of suitable donor organs (5, 6).

Following the first publication of brain death criteria in 1968 (7–9), cardiac transplantation has been performed almost exclusively with organs procured from donors that have been declared dead based on neurologic criteria [donation after brain death (DBD), Figure 1A]. In addition to improving the utilization rate of DBD hearts, exploration of alternative donor sources is warranted to mitigate the growing disparity between the number of eligible transplant recipients and available organs.

**Figure 1:**
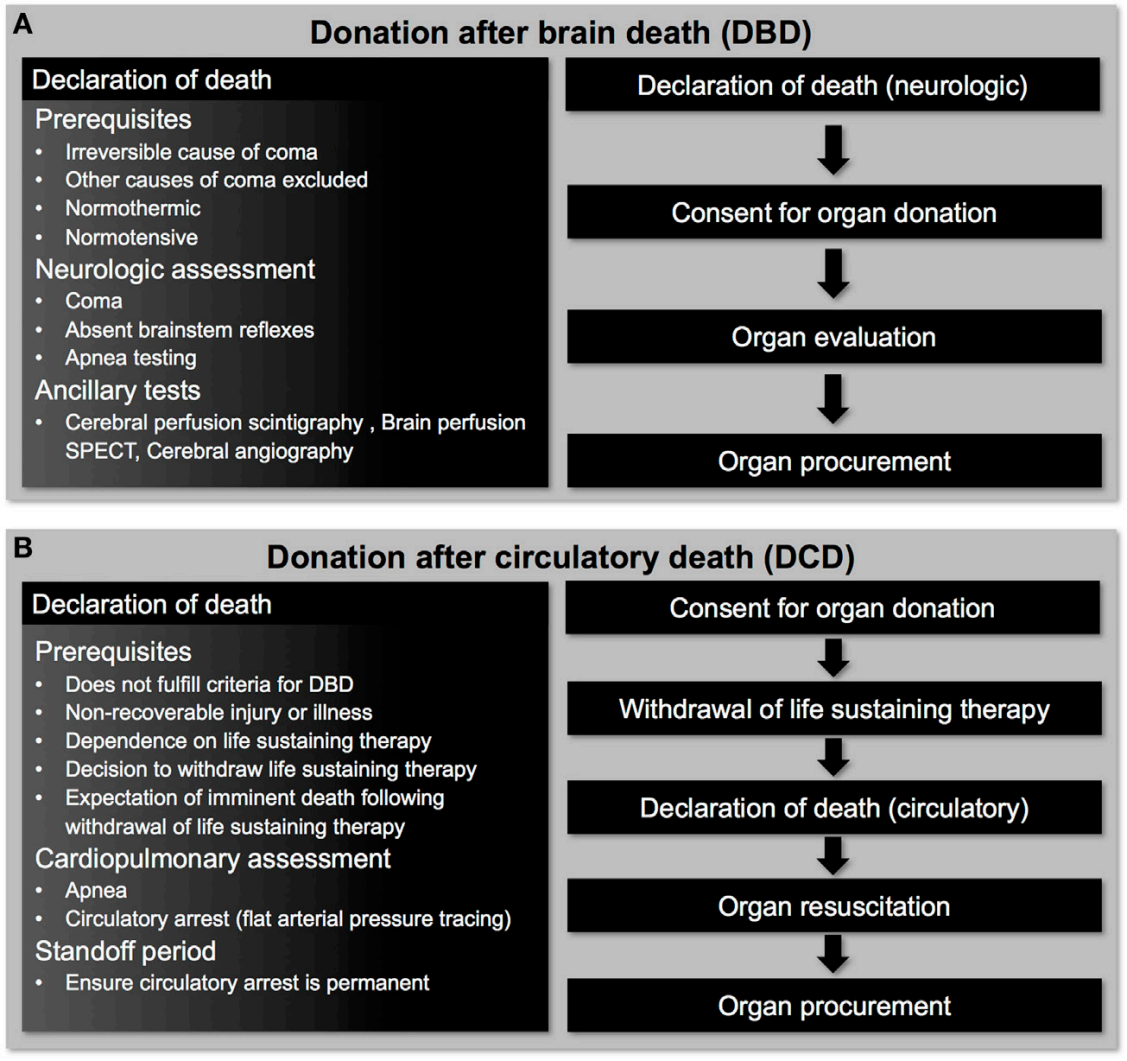
Pathways for deceased organ donation. **(A)** Patients donating organs after brain death have intact cardiorespiratory function that allows donor heart evaluation to be undertaken before organ procurement. **(B)** Patients donating organs after circulatory death have suffered a hypoxemic cardiac arrest following withdrawal of life-sustaining therapy and donor heart evaluation can only be undertaken after organ resuscitation has occurred.

## Donation after Circulatory Death (DCD)

Donation after circulatory death describes the procurement of organs from donors that have been declared dead based on circulatory criteria (Figure 1B) (10). Early transplant programs utilized organs from DCD donors, including the first heart transplants performed by Christiaan Barnard (11, 12); however, this practice was largely abandoned following the acceptance of brain-death criteria (7–9). Today, a critical shortage of suitable organs from DBD donors and the expansion of DCD programs have prompted a renewed interest in DCD heart transplantation. The DCD pathway for organ donation is increasing in many countries around the world, and in some countries accounts for more than one-third of all deceased organ donation (13–16). DCD accounted for 42% of kidney, 19% of liver, and 19% of lung transplants from deceased donors in the UK in 2012 (17), while DCD accounted for 21% of all deceased organ donation in Canada in 2015 (18).

**Figure 2:**
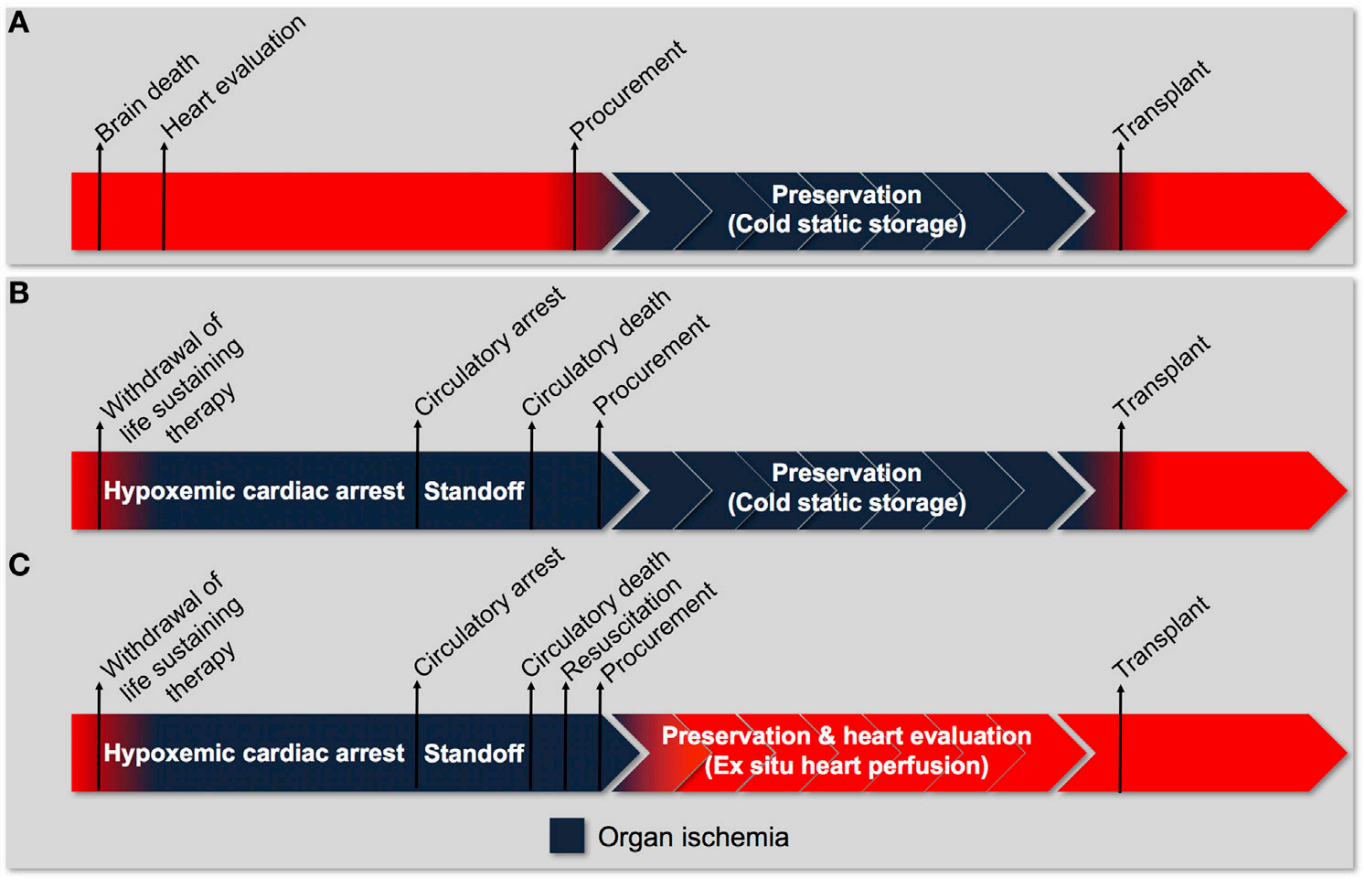
Process of heart transplantation. **(A)** The traditional approach to transplantation of a heart procured from a donation after brain death donor. Donor heart evaluation is carried out in the donor with intact cardiorespiratory function. Viable organs are arrested with a cardioplegic solution and stored in a profoundly hypothermic state (cold-static storage) until transplantation. Organ ischemia is limited to the time between procurement and transplantation (cold ischemic time). **(B)** The traditional approach to transplantation if it were utilized for a heart procured from a donation after circulatory death donor. The donor progresses to circulatory arrest following withdrawal of life-sustaining therapy (WLST). An ethically mandated standoff period must then be observed before circulatory death can be declared. Consequently, the heart has sustained a significant warm ischemic insult before organ procurement can proceed. Subsequent preservation using cold-static storage subjects the heart to an additional cold ischemic injury and does not provide an opportunity for organ resuscitation and evaluation. The traditional approach is unlikely to facilitate successful transplantation of hearts donated after circulatory death. **(C)** The tailored approach to transplantation of a heart procured from a donation after circulatory death donor. Following WLST and declaration of circulatory death, the heart is resuscitated using an approach tailored to minimize ischemia-reperfusion injury. The heart is then preserved using *ex situ* heart perfusion, which minimizes exposure to cold ischemia and facilitates organ evaluation. Organ ischemia can be limited to the time between WLST and organ resuscitation (warm ischemic time).

The Maastricht classification system is used to describe four different categories of DCD donors according to the circumstances of the donor’s death (Table 1) (19, 20). Uncontrolled DCD (Maastricht Category I, II, and IV) refers to donors having suffered an unexpected cardiac arrest and unsuccessful resuscitation. Controlled DCD (Maastricht Category III and IV) refers to donors that undergo a planned withdrawal of life-sustaining therapy (WLST) and progression to circulatory arrest. Experimental and clinical transplantation of DCD hearts have been restricted to Maastricht Category III donors. These donors typically have a non-recoverable neurologic injury, are dependent on advanced life support therapies, but do not meet formal brain-death criteria. If ongoing medical care is deemed futile and a decision to WLST is made, consent for organ donation may be obtained. In this scenario, life-sustaining therapies are withdrawn and palliative care is provided according to institutional practices. The patient is monitored for progression to apnea and circulatory arrest, which is declared when a pulse pressure is no longer present on an arterial pressure tracing (mechanical asystole) (20). However, some jurisdictions require progression to electrical asystole before circulatory arrest can be declared (21). An ethically mandated 5-min standoff period (may vary from 2 to 20 min depending on institutional protocols) is then observed before circulatory death is declared and organ procurement can proceed (Figures 2B,C) (21). Therefore, the diagnosis of death is based on the cessation of cardiorespiratory function (20). This differs in many respects from DBD, where the declaration of death is based on neurologic criteria in a donor that still has intact cardiac function.

**Table 1:** Modified Maastricht classification of donation after circulatory death (19, 20).

Uncontrolled	Description
Category 1	*Found dead*
	Sudden unexpected circulatory arrest without any attempt of resuscitation by a medical team
	Category IA: out-of-hospital, Category IB: in-hospital

Category II	*Witnessed cardiac arrest*
	Sudden unexpected irreversible circulatory arrest with unsuccessful resuscitation by a medical team
	Category IIA: out-of-hospital, Category IIB: in-hospital

Category IV	*Cardiac arrest while brain dead*
	Sudden circulatory arrest after brain-death diagnosis during donor management but prior to planned
	Organ retrieval; donation proceeds after unsuccessful resuscitation by a medical team

**Controlled**	**Description**

Category III	*Withdrawal of life-sustaining therapy (WLST)*
	Planned WLST and expected circulatory arrest

Category IV	*Cardiac arrest while brain dead*
	In countries where legislation does not accept brain death criteria or patient will never meet the neurologic criteria for the diagnosis of brain death, the procedure for this potential DBD can be converted to a DCD

Donation after circulatory death hearts experience a period of warm ischemia during the progression to circulatory arrest and declaration of death (Figures 2B,C), the duration of which is an important criterion in the selection of hearts for transplantation (22, 23). The warm ischemic time (WIT) officially refers to the time between WLST and organ reperfusion (20); however, some Maastricht category III donors maintain a stable cardiorespiratory status for a prolonged time before eventual progression to circulatory arrest. Thus, the functional warm ischemic time (FWIT) refers to the time from when the systolic blood pressure decreases below 50 mmHg for at least 2 min until organ reperfusion (20). The FWIT is meant to provide a more accurate estimate of the actual ischemic injury sustained by donor organs following WLST. However, insufficient end-organ oxygen delivery and the onset of organ ischemia (evidenced by rising systemic lactate concentrations) may occur before a significant decline in blood pressure has occurred (24). Oxygen desaturation may be a more sensitive indicator of end-organ ischemia and should be included in the FWIT definition (24).

### Potential Impact of DCD in Heart Transplantation

Multiple authors have attempted to describe the potential impact of DCD on the numbers of heart transplants that could be performed. Noterdaeme et al. examined deceased donor data from a single center in Belgium (25). Over a 6-year period, there were 247 deceased donors, 28% of whom were Maastricht Category III donors. The authors applied the same inclusion criteria used for DBD heart donors, and required that the WIT not exceed 30 min. This approach identified 8 potential DCD hearts with a WIT of 15.1 ± 0.5 min. During the same time period, 82 patients were listed for heart transplantation, 53 were transplanted, 9 were still waiting, 11 were removed from the list, and 9 died while waiting. The authors conclude that the transplantation of eight additional hearts could have significantly reduced the waiting list mortality and increased heart transplant activity by 15%. Messer et al examined 3,073 DCD donors referred over a 3-year period in the UK and found 149 (5%) to be suitable heart donors (26), which could have grown transplant activity by 30%. In the US and Australia, DCD heart transplantation could increase transplant activity by 4–17% (27–29). Overall, DCD heart transplantation has the potential to significantly increase the annual transplant volume in many countries with an attendant reduction in waitlist mortality.

## DCD Heart Transplantation

The approach to DBD heart transplantation displayed in Figure 2A represents the current standard of care for the procurement and preservation of DBD hearts. Following the declaration of brain death, heart function is evaluated to determine suitability for donation. At the time of organ procurement, the donor has intact cardiac function and the heart is not ischemic. Hearts are electromechanically arrested using a cold, hyperkalemic cardioplegic solution and undergo cold-static storage until they are transplanted. The DBD heart is only exposed to ischemia in the time between organ procurement and transplantation (Figure 2A).

**Figure 3:**
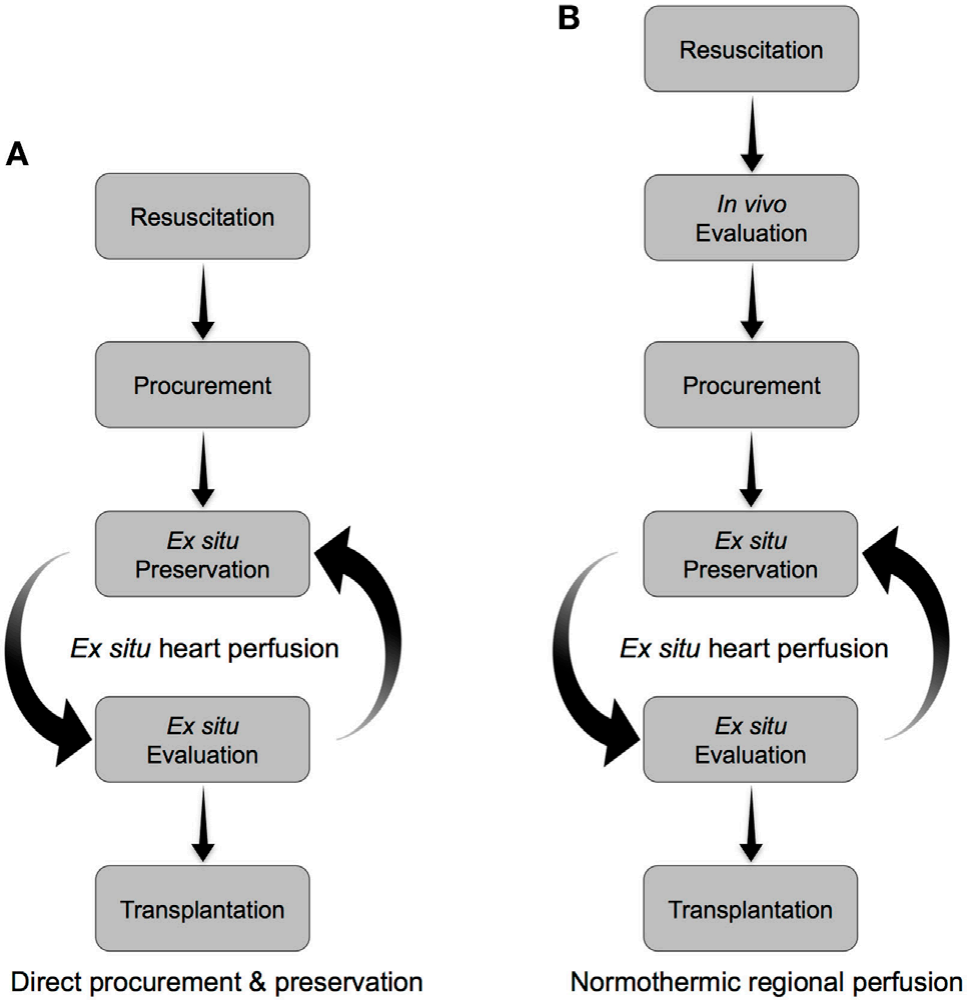
Alternative approaches to the resuscitation, preservation, and evaluation of hearts donated after circulatory arrest. **(A)** Direct procurement and preservation. Hearts are resuscitated with a cardioplegic solution tailored to minimize ischemia-reperfusion injury, then preserved *ex situ* in a normothermic beating state. Organ evaluation is carried out during *ex situ* preservation to identify viable organs for transplant. **(B)** Normothermic regional perfusion. Hearts are resuscitated *in vivo* on veno-arterial extracorporeal membrane oxygenation (ECMO). The donor is subsequently weaned from ECMO, *in vivo* assessments of heart function are carried out, and then viable organs are procured and preserved *ex situ* in a normothermic beating state until transplant. Supplementary organ evaluation can be carried out during *ex situ* preservation.

Simply applying the standard DBD approach for heart procurement and preservation to the DCD context is unlikely to allow for adequate resuscitation of the DCD heart to provide a viable organ (Figure 2B). The DCD heart has already sustained significant warm ischemia following WLST in the donor (during the progression to circulatory arrest and the warm ischemic standoff period) and would not tolerate the additional ischemic injury during cold-static storage. Therefore, an approach tailored specifically to the DCD context is required to facilitate successful transplantation (Figure 2C). Such an approach must include (1) organ resuscitation at the time of procurement to minimize the detrimental effects of warm ischemia following donor extubation, (2) a preservation strategy that minimizes additional ischemic injury and provides an opportunity for organ reconditioning, and (3) the ability to assess organ viability prior to transplantation (Figures 2C and 3).

### Part 1: DCD Heart Resuscitation

#### Ischemia–Reperfusion Injury

Understanding the physiologic impact of donor extubation and warm ischemia on the DCD heart is fundamental to developing a successful resuscitation strategy (24). After extubation, the DCD heart is forced to function in an increasingly hypoxemic environment while attempting to maintain systemic oxygen delivery (24). Progressive hypoxemia and hypercarbia cause constriction of the pulmonary vasculature and distention of the right ventricle. These changes prompt a catecholamine surge and a transient hyperdynamic circulatory phase; however, myocardial energy stores are rapidly depleted, cardiac output declines, and the donor progresses to circulatory arrest (24). The donor heart remains in a warm, distended, and ischemic state during the ethically mandated standoff period (24); therefore, at the time of organ procurement the DCD heart has withstood exposure to a catecholamine surge and sustained significant ischemic injury (Figures 2B,C).

**Figure 4:**
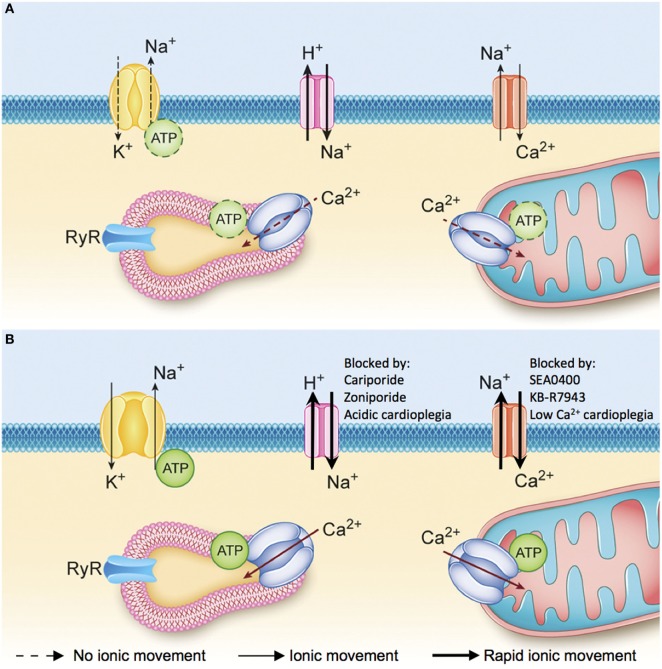
**(A)** Ionic changes during ischemia. Anaerobic metabolism results in the production of hydrogen ions that activate the sodium–hydrogen exchanger and the accumulation of sodium ions inside the myocyte. The sodium–potassium ATPase is not able to extrude the excess sodium ions and maintain the normal membrane potential due to a lack of available adenosine triphosphate (ATP). Consequently, as ischemia progresses there is an accumulation of sodium and hydrogen ions inside the myocyte and depolarization of the membrane potential. **(B)** Ionic changes during reperfusion. Reperfusion washes out the hydrogen ions that have accumulated in the interstitial space and creates a large gradient for sodium–hydrogen exchange. The influx of sodium ions into the myocyte during early reperfusion forces the sodium–calcium exchanger (NCX) to function in reverse mode and import calcium ions across the sarcolemma. Intracellular ionic homeostasis cannot be restored until the sodium-potassium ATPase is able to reestablish the resting membrane potential and normal intracellular sodium levels, which will allow the NCX to return to a forward mode of operation and extrude excess calcium from the cytoplasm [adapted with permission (34)].

**Figure 5:**
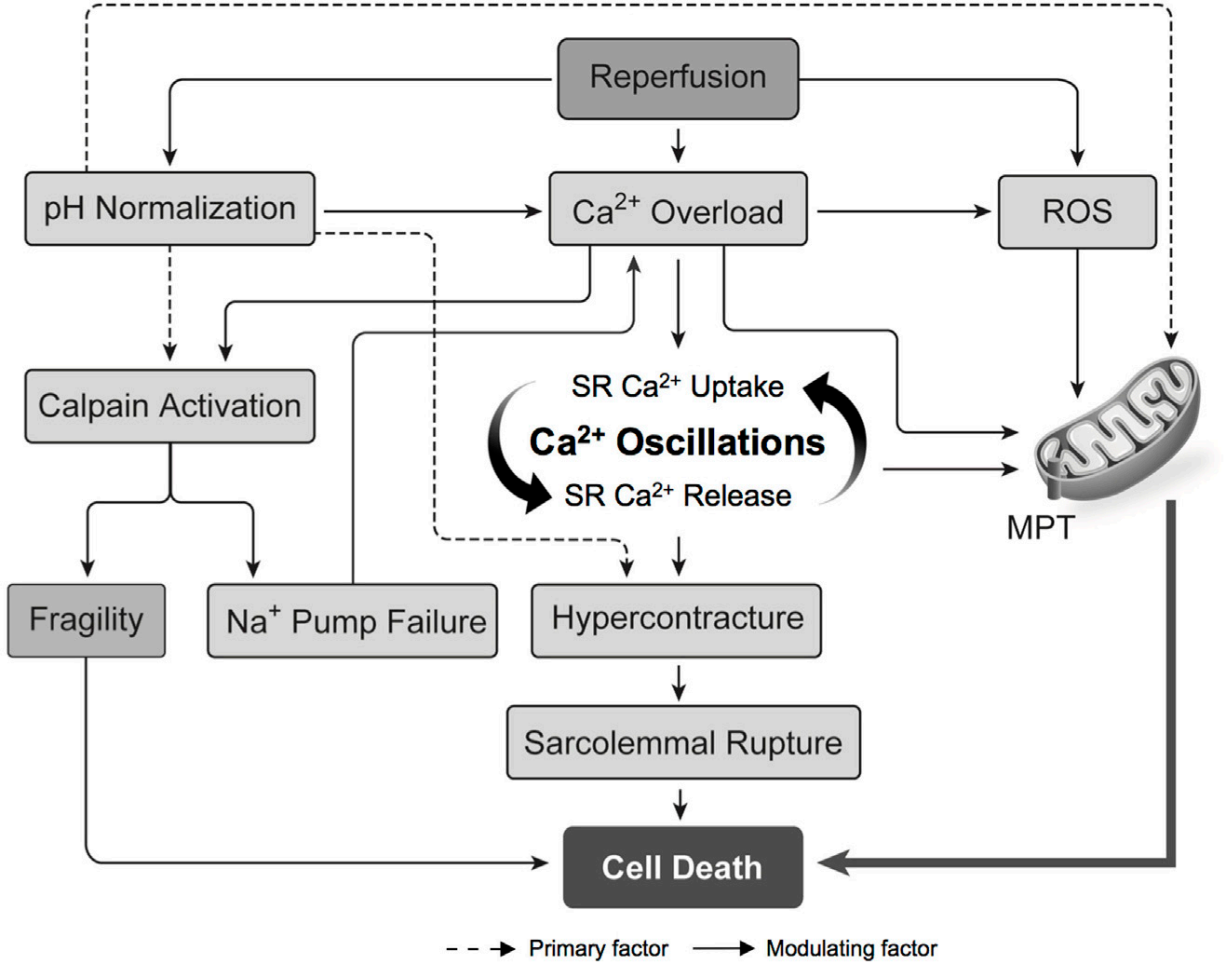
Pathogenesis of ischemia–reperfusion injury. Intracellular calcium overload and the production of ROS cause opening of the MPT pore and the propagation of cell death. The normalization of intracellular pH during reperfusion is an important modulating factor in the pathogenesis of ischemia–reperfusion injury. Abbreviations: MPT, mitochondrial permeability transition; ROS, reactive oxygen species; SR, sarcoplasmic reticulum [adapted with permission (31)].

The ischemic injury sustained by the DCD heart results in the depletion of adenosine triphosphate (ATP) stores and anaerobic metabolism, which cause intracellular acidosis, activation of the sodium–hydrogen exchanger (NHE), and sodium influx into the myocyte (30, 31). The sodium-potassium ATPase normally functions to extrude sodium ions entering the myocyte *via* the NHE. In the DCD context, however, intracellular acidosis develops concurrently with the depletion of ATP stores. The combined effect of increased NHE activity and inhibition of the sodium–potassium ATPase produces a pathological accumulation of intracellular sodium (Figure 4A) (31, 32). Subsequent reperfusion at the time of organ procurement rapidly normalizes the extracellular pH and creates a large hydrogen ion gradient across the plasma membrane that causes further sodium influx *via* the NHE (33). This increase in intracellular sodium forces the sodium–calcium exchanger (NCX) to function in reverse mode and import calcium ions across the sarcolemma (Figure 4B). The resultant intracellular calcium overload propagates myocyte death through the development of hypercontracture, generation of reactive oxygen species (ROS), activation of the mitochondrial permeability transition (MPT) pore, and initiation of apoptotic pathways (Figure 5) (30, 31, 33). Limiting the severity of ischemia-reperfusion injury (IRI) at the time of organ procurement represents the cornerstone of DCD heart resuscitation.

#### Approach to DCD Heart Resuscitation

With the aim of minimizing IRI and optimizing functional recovery, two methods of DCD heart resuscitation have emerged and have been successfully utilized in the clinical context (Table 4) (35). The first approach has been termed Direct Procurement and Perfusion (DPP). This approach involves delivery of a cardioplegic solution during organ procurement that is designed to promote ischemic post-conditioning and limit the detrimental effects of IRI. With this approach, a rapid cardiectomy is performed, the heart is connected to an *ex situ* heart perfusion (ESHP) device and preserved in a normothermic and beating state until eventual transplantation (Figure 3A) (22, 36). The second approach is Normothermic Regional Perfusion (NRP) (35). Following declaration of circulatory death, a median sternotomy is performed and the cerebral circulation is isolated (a clamp is placed across the arch vessels). With the brain excluded from the circulation, the donor is placed on veno-arterial extracorporeal membrane oxygenation (ECMO) and reperfused for 60 min. The donor is subsequently weaned from ECMO, which facilitates the assessment of donor heart function *in vivo*. Viable organs are then arrested with a traditional cardioplegic solution, connected to an ESHP device, and preserved in a normothermic, beating state until transplantation (Figure 3B) (23, 35).

### Part 1A: DCD Heart Resuscitation: DPP

#### Initial Reperfusion Solution

At the time of organ procurement, the DCD heart is energy deplete and vulnerable to the influx of sodium and calcium upon reperfusion. The reactivation of ATP production in the calcium-overloaded myocyte after prolonged ischemia can propagate calcium oscillations and the development of hypercontracture (Figure 5) (37, 38). However, initial reperfusion with a cardioplegic solution inhibits myocardial contraction at the onset of re-oxygenation. This facilitates the repletion of myocardial energy stores and the restoration of intracellular calcium homeostasis before activation of the myofibrillar contractile unit, thereby preventing hypercontracture (39, 40). Previous research has demonstrated that hearts subjected to a period of ischemia exhibit better functional recovery when reperfused with a cardioplegic solution compared to reperfusion with unmodified blood (41–44). Therefore, initial reperfusion of the DCD heart should maintain cardiac arrest to provide an opportunity for the restoration of intracellular ion homeostasis and limit hypercontracture.

#### Cardioplegia Composition

In 1986, Murry et al. first demonstrated that repetitive periods of brief ischemia could protect the myocardium from a subsequent period of prolonged ischemia and established the concept of ischemic pre-conditioning (45). In 2003, Zhao et al. demonstrated that repeated cycles of ischemia and reperfusion following a prolonged index ischemic insult (ischemic post-conditioning) could attenuate reperfusion-induced myocardial injury to the same extent achieved with ischemic pre-conditioning (46). Subsequent research has demonstrated that common molecular pathways are involved in these two processes (47). These landmark discoveries have tremendous implications for DCD heart transplantation because the delivery of therapeutics to the donor prior to the declaration of death (ischemic pre-conditioning) is ethically prohibited in the majority of jurisdictions. However, the activation of ischemic post-conditioning pathways at the time of organ procurement provides an opportunity to mitigate IRI and resuscitate the DCD heart. The therapeutic window is narrow and optimal success is realized in the first minutes of organ reperfusion (48); therefore, enhancing the composition of the initial reperfusion cardioplegia to activate ischemic post-conditioning pathways and inhibit mediators of IRI is the primary focus of DCD heart resuscitation.

The activation of prosurvival kinases at the time of reperfusion confers powerful cardioprotection against IRI through inhibition of the MPT pore, and activation of the reperfusion injury salvage kinase (RISK) and survivor activating factor enhancement (SAFE) pathways (47, 49, 50). The delivery of erythropoietin, adenosine, and insulin during reperfusion has been shown to activate the RISK pathway and mitigate myocardial IRI (49, 51, 52). Erythropoietin-supplemented cardioplegia has also been shown to activate the SAFE pathway and inhibit mitochondrial permeability pore transition (53–55). Additionally, insulin causes vasodilation, inhibits apoptosis, limits the inflammatory response, and reduces ROS generation (56). Adenosine inhibits apoptosis through upregulation of the antiapoptotic protein Bcl-2 and has anti-inflammatory properties that attenuate neutrophil infiltration into endothelial cells and ROS production (57–63). The nitric oxide donor glyceryl-trinitrate has been shown to activate prosurvival kinases and Bcl-2 (64). Overall, a variety of pharmacologic activators of the RISK and SAFE pathways confer protection against IRI and hold great promise in optimizing DCD heart resuscitation.

The influx of sodium and calcium into the myocyte play a central role in the pathogenesis of IRI. Reperfusion at the time of organ procurement normalizes the extracellular pH, and creates a large hydrogen ion gradient across the plasma membrane that causes sodium influx *via* the NHE and calcium influx *via* reverse NCX (Figure 4B). Inhibiting these ionic fluxes early in the reperfusion period represents an important opportunity to limit hypercontracture and activation of the MPT pore (Figure 5) (49).

The cardioprotective effects of NHE inhibition in IRI are well documented. Cariporide has been shown to limit myocardial IRI in clinical trials (65–67) and translational animal models of DCD heart transplantation (32, 68–70). Zoniporide is a recently developed NHE inhibitor that possesses greater potency and selectivity toward the cardiac NHE (71), and has potent cardioprotective effects through activation of prosurvival kinase pathways and inhibition of apoptosis (72). Unfortunately, NHE inhibitors are no longer available for clinical use and enthusiasm for further development has been limited by the results of the Expedition trial (67). However, drug delivery in the context of DCD heart resuscitation is isolated to the heart in a deceased donor and the potential detrimental impact on other organ systems is irrelevant. Therefore, NHE inhibitors may still have an important role in DCD hearts resuscitation. Alternatively, delaying pH normalization at the onset of reperfusion by delivering an acidic cardioplegic solution inhibits NHE, and limits calcium overload and IRI (Figures 4 and 5) (73–75). We have recently shown that initial reperfusion with a moderately acidic solution optimizes the functional recovery of DCD hearts (34). Therefore, modifying the cardioplegic solution composition may provide an avenue to realize the clinical benefit of NHE inhibition without relying on pharmacologic inhibitors that are not currently available for clinical use.

Pharmacologic inhibitors of reverse-mode NCX have also been investigated as a means of minimizing calcium influx into the myocyte during reperfusion (Figure 4). NCX inhibitors minimize calcium overload, hypercontracture, infarct size, and contractile dysfunction following ischemia in animal models (76–80), and may be more cardioprotective than NHE inhibitors (81). While there are no clinically approved NCX inhibitors at present, calcium influx during reperfusion can be limited if the initial reperfusion solution is rendered hypocalcemic. This serves to minimize the calcium gradient that favors reverse NCX activity during early reperfusion, thereby limiting calcium overload (82–86). We have recently demonstrated that initial hypocalcemic reperfusion optimized the functional recovery of DCD hearts (34). Therefore, utilizing a hypocalcemic cardioplegic solution may provide a simple avenue achieve NCX inhibition and minimize IRI.

Conventional cardioplegic solutions rely on hyperkalemia to depolarize the membrane potential and achieve diastolic arrest; however, membrane depolarization is associated with an increase in intracellular sodium *via* non-activating sodium currents that may exacerbate calcium overload during reperfusion (72). Alternatively, normokalemic adenosine-lidocaine cardioplegic solutions have been proposed, in which lidocaine blockade of sodium fast channels causes a diastolic arrest and adenosine maintains a polarized membrane potential (87–89). Ischemic rat hearts reperfused with an adenosine-lidocaine cardioplegia exhibit improved myocardial function compared to traditional hyperkalemic cardioplegia (88). Similarly, polarized arrests using potassium channel openers minimize calcium overload and improve myocardial function (90–92). Mohri et al. have applied this concept in a large animal model of DCD heart transplantation and demonstrated improved posttransplant outcomes in hearts treated with a potassium channel opener (93). Further, we have utilized normokalemic adenosine-lidocaine based solutions in our translational models of DCD heart transplantation (34, 94, 95). Further research is required to determine the role of such alternative cardioplegic solutions in clinical DCD heart transplantation.

A wide variety of strategies have demonstrated efficacy in mitigating IRI; however, it is likely that the delivery of a cardioplegic solution containing a cocktail of complementary pharmacologic post-conditioning agents that target a variety of pathways involved in the pathogenesis of IRI will further improve DCD heart resuscitation. For example, the synergistic beneficial effects of glyceryl-trinitrate, erythropoietin, and zoniporide have been demonstrated in animal models of DCD transplantation (96, 97). We have utilized a cardioplegic solution containing the ischemic post-conditioning agents, adenosine and insulin, and rendered the solution acidic and hypocalcemic to minimize calcium overload during reperfusion (34). ROS scavengers have been shown to limit IRI in DCD hearts (98); however, ROS signaling in the early reperfusion period is essential for the activation of post-conditioning pathways and the role of ROS scavengers in DCD heart resuscitation requires further research (99–101). Calpain inhibitors and MPT pore inhibitors may represent other pharmacologic strategies to further optimize DCD heart resuscitation (102, 103). Further research in this area will undoubtedly contribute to improved posttransplant outcomes in the future.

#### Cardioplegia Delivery

Optimizing the conditions of the initial reperfusion may further improve DCD heart resuscitation. Initial cardioplegic reperfusion provides an opportunity to restore myocardial energy stores and intracellular ion homeostasis prior to myocardial contraction; however, hypothermia markedly lowers the activity of the ion pumps that restore intracellular ionic homeostasis (104–106). The delivery of hypothermic cardioplegia at the time of organ procurement is standard practice when procuring hearts from DBD donors, to minimize metabolic demands during the subsequent period of cold-static storage (Figure 2A). In the context of DCD; however, the benefit of inducing a profoundly hypothermic state in the short period of time between procurement and initiation of normothermic ESHP may be outweighed by the negative impact of hypothermia on the reparative processes essential for resuscitation of the DCD heart (95).

Previous work has demonstrated that delivery of warm cardioplegia following ischemia improves functional recovery (44, 107–109). These results have been confirmed in clinical trials of patients undergoing cardiac surgery, which demonstrated that terminal warm cardioplegia limits myocardial injury and improves function postoperatively (110, 111). Since DCD hearts have sustained significant ischemic injury, application of the terminal warm cardioplegia concept to the initial reperfusion of DCD hearts may optimize their resuscitation (41). Translational experiments in animal models of DCD have advocated for the avoidance of profound hypothermia during the initial reperfusion (41, 94, 98, 112–114), and we recently confirmed that the avoidance of profound hypothermia during initial reperfusion minimizes injury and improves the functional recovery (95).

Interventions to limit IRI must be administered at the onset of reperfusion to be effective; however, the duration of the cardioplegic reperfusion is another variable that impacts DCD heart resuscitation. The initial reperfusion must be of sufficient duration to facilitate repletion of myocardial energy stores, restore ionic homeostasis, and activate ischemic post-conditioning pathways. Cohen et al. have demonstrated that initial reperfusion with an acidic solution for 1 min was not protective; however, when the reperfusion was extended to 2 min it afforded protection against IRI equivalent to that achieved with post-conditioning protocols (115). In contrast, studies have demonstrated that initial reperfusion with an inhibitor of GSK-3β must be extended over 15 min to be effective (116) and initial reperfusion with adenosine required an infusion extended over 40 min to achieve a post-conditioning effect (117). Osaki et al. found that posttransplant outcomes were optimized when initial reperfusion of DCD hearts with a blood-based cardioplegia at 20°C was continued for 20 min (39). These results suggest that the optimal initial reperfusion duration may depend on the composition of the reperfusion solution, the conditions of its delivery, and the means by which activation of the ischemic post-conditioning pathways occur.

### Part 1B: DCD Heart Resuscitation: NRP

Current protocols for NRP involve reperfusion with donor blood following initiation of veno-arterial ECMO (35). Donor blood at the time of reperfusion may have beneficial properties that would facilitate DCD heart resuscitation. Following donor extubation a mixed respiratory and metabolic acidosis develops (24). As previously discussed, initial acidic reperfusion of ischemic myocardium limits IRI (Figure 5) (73–75). The DCD heart also has reduced antioxidant capacity in the early reperfusion period, and exposure to high oxygen partial pressures may propagate ROS production (118). Therefore, initial reperfusion with the hypoxemic and acidic blood of the DCD donor may actually be beneficial. The energy substrates and buffers that exist in donor blood may facilitate restoration of myocardial energy stores. In these regards, NRP may serve to resuscitate the DCD heart. Proponents also believe that it provides a more expeditious reperfusion and minimizes the WIT compared to DPP (35). A potential downside of the NRP method, however, is the high levels of catecholamines and pro-inflammatory cytokines that are present within the DCD donor that may have detrimental effects on myocardial resuscitation (119, 120).

Despite divergent approaches to DCD heart resuscitation, (Table 4) both DPP and NRP protocols have been successfully implemented in clinical programs with excellent results (22, 23). Further research is required to determine if one approach is superior to the other.

### Part 2: DCD Heart Preservation

Cold-static storage represents the current standard method of DBD heart preservation prior to transplantation (Figure 2A); however, the DCD heart has withstood a significant ischemic insult and exposure to incremental cold ischemia during the preservation interval is unlikely to facilitate successful organ resuscitation (Figure 2B) (121). It is also necessary to confirm organ viability prior to transplantation given the severity of injury sustained by the DCD heart; however, static storage under hypothermic conditions prohibits such assessments from being undertaken (122). ESHP has been investigated as a means to minimize exposure to cold ischemia and support aerobic metabolism during organ preservation, thereby extending the safe preservation interval (39, 122–131). Two methods of ESHP for DCD heart preservation have emerged: (1) normothermic perfusion and (2) hypothermic perfusion.

### Part 2A: DCD Heart Preservation: Normothermic Perfusion

Normothermic ESHP has been shown to maintain aerobic metabolism and limit exposure to cold ischemia during organ preservation. It also facilitates the delivery of pharmaceuticals to recondition dysfunctional organs, including stem cell and gene therapies (132–136). Finally, normothermic ESHP provides an opportunity to perform metabolic and functional assessments during the preservation interval in order to identify organs that are suitable for transplantation (Figures 2C and 3) (22, 137, 138).

The Transmedics organ care system (OCS) is the only clinically available ESHP device, and the results of the *Ex vivo* perfusion of donor hearts for human heart transplantation (PROCEED II) trial that describe the clinical outcomes of standard criteria DBD hearts preserved using the OCS have been recently published (137). The OCS is primed with 1.5 L of leukocyte-depleted whole blood obtained from the donor during organ procurement. This is combined with 0.5 L of proprietary priming solution containing a physiologic salt solution, heparin (10,000 IU), mannitol (12.5 g), methylprednisolone (250 mg), multivitamins (1 U), insulin (80 IU), ciprofloxacin (100 mg), and cefazolin (1 g). During perfusion, a maintenance solution containing dextrose, amino acids, adenosine, and epinephrine, is titrated to achieve a desired aortic perfusion pressure and coronary blood flow. The perfusate solution circulates through an oxygenator and heat exchanger such that the coronaries are perfused with an oxygenated solution. The left ventricle is preserved in an unloaded and beating state; therefore, assessments of organ viability are limited to metabolic profiles (lactate metabolism) and perfusion parameters (137). The PROCEED II trial randomized 130 transplant patients to receive a standard criteria donor heart preserved with either the OCS or using conventional cold-static storage. The primary endpoint of 30-day patient and graft survival in the OCS group [63/67 patients (94%)] was non-inferior compared to the cold-static storage group [61/63 patients (97%)] (137); however, five standard criteria donor hearts that were randomized to the OCS group were deemed non-viable based on their lactate profile and not ultimately transplanted. These hearts were not included in the final analysis and should be considered in the interpretation of the study results (139).

Donor heart preservation using ESHP has been utilized in numerous pre-clinical studies of DCD heart transplantation (39, 94, 97, 98, 140–142). Notably, Iyer et al. demonstrated the impact of ESHP in a large animal model of DCD heart transplantation (140). Donor hearts were assigned to conventional cold-static storage or preservation using the OCS. None of the hearts preserved using cold-static storage could be weaned from cardiopulmonary bypass, while all the hearts deemed viable following perfusion on the OCS were successfully transplanted. These results underscore the importance of minimizing DCD heart exposure to cold ischemia during the preservation interval and the utility of ESHP in facilitating successful transplantation (Figures 2C and 3). The OCS has been subsequently utilized in clinical DCD heart transplantation programs in Australia and UK (23, 137).

Despite the successful clinical application of donor heart preservation using the OCS, many questions regarding the optimal conduct normothermic ESHP remain unanswered (139). The optimal perfusate composition (oncotic pressure, hematocrit, metabolic substrates, cardioprotective additives, etc.), coronary perfusion pressure, and perfusion temperature are largely unknown. Much research is required to optimize the conduct of ESHP and realize the potential of this technology in clinical transplantation.

The OCS utilizes a whole blood-based perfusate to ensure adequate myocardial oxygen delivery. Preservation using a whole blood-based perfusate may improve donor heart preservation compared to a solution in which the plasma component has been removed. This observation may be related to the antioxidant and anti-inflammatory properties of albumin and other plasma proteins, and the metabolic substrates present in donor plasma (143). Previous research has demonstrated that an oxygen carrier is required to meet the metabolic demands of a working heart at normothermia; however, further studies are required to determine the optimal hemoglobin concentration (143).

The development of myocardial edema is common during ESHP, which may cause diastolic dysfunction and limit the safe preservation interval (144). The OCS prime solution is supplemented with methylprednisolone in order to minimize myocardial edema. Donor heart exposure to extracorporeal circulation during ESHP has been shown to elicit an inflammatory response, with a 60-fold increase in pro-inflammatory cytokines observed over a 5-h preservation interval (145). Methylprednisolone may limit this inflammatory response and minimize the development of myocardial edema (145). Oshima et al. have also demonstrated that the suppression pro-inflammatory cytokines during ESHP improves posttransplant myocardial function (146).

Another important variable impacting the development of myocardial edema is the perfusate solution oncotic pressure. The OCS priming solution contains mannitol as an oncotic agent; however, the oncotic pressure of this solution has not been reported. We have utilized a perfusate solution comprised of STEEN Solution™ (XVIVO Perfusion, Goteborg, Sweden) and whole donor blood (34, 95, 138, 143). STEEN solution™ is a buffered extracellular-type salt solution containing human serum albumin and dextran 40 for oncotic pressure, and when combined with donor blood the oncotic pressure of the perfusate solution is 33 ± 1 mmHg (143). This is supra-physiologic compared to the normal human oncotic pressure of 25 mmHg (147); however, normal hearts may still gain up to 12% of their initial heart weight and DCD hearts up to 24% over a 6-h preservation interval (95). Sufficient oncotic pressure must be maintained to minimize the development of myocardial edema (123), yet neither the optimal oncotic pressure or impermeant (albumin, mannitol, lactobionic acid, dextran, hydroxyethyl starch, succinylated gelatin) have been established.

The coronary perfusion pressure may also impact the development of myocardial edema. Inadequate perfusion pressure may compromise myocardial oxygen delivery, while excessive pressure may damage endothelial cells (148). The target OCS perfusion pressure is 65–90 mmHg (149); however, we have observed that myocardial energy stores can be maintained with aortic pressures as low as 40 mmHg (143). Coronary perfusion pressure is delivered in a pulsatile fashion on the OCS, and previous authors have demonstrated that biologically variable perfusion may reduce myocardial edema (121). Further research is required to determine the optimal coronary perfusion pressure during ESHP.

Previous studies have demonstrated that myocardial function declines in a linear fashion during ESHP, even when normal hearts undergo prolonged perfusion (95, 143). Such a functional decline limits the safe preservation time and the potential of resuscitating dysfunctional hearts. Fatty acids and carbohydrates represent the primary metabolic substrates for ATP production under normal conditions, and the respective contribution of each substrate to oxidative metabolism is tightly regulated (150). Pathological states can alter these pathways, and optimizing metabolic substrate utilization during ESHP may dramatically improve the preservation of myocardial function. Current ESHP protocols include amino acids, glucose, and insulin as exogenous substrates (137); however, there is a paucity of research investigating the optimal substrate provision. Interestingly, previous authors have demonstrated that the oxidation of glucose during ESHP is limited, while pyruvate is rapidly incorporated into the tricarboxylic acid cycle and improves myocardial function (151, 152). While amino acids are not generally used as primary substrates for energy production, they play important roles in the intermediary metabolism of the cardiomyocytes and may regulate substrate utilization (118). Free fatty acids are rapidly depleted during ESHP (153); however, the impact of exogenous supplementation on myocardial function has not been investigated. It appears that the perfusate substrate composition may impact myocardial energy metabolism during ESHP and the preservation of donor heart function. Further research in this area may dramatically improve donor heart preservation during ESHP.

### Part 2B: DCD Heart Preservation: Hypothermic Perfusion

Hypothermic perfusion has been investigated as an alternative means of supporting aerobic metabolism and minimizing DCD heart exposure to cold ischemia during the preservation interval. The myocardial oxygen demand of a hypothermic and arrested heart is only 0.14 mL/100 g/min (154), and oxygen delivery to meet myocardial energy requirements in this state can be achieved with an oxygenated crystalloid perfusate solution delivered at approximately 20 mL/min and an aortic pressure of 5 mmHg (155, 156). Consequently, hypothermic perfusion can be achieved using a simple and less expensive perfusion apparatus compared to the technology required to perfuse a normothermic beating heart with a blood-based perfusate solution (155, 156).

Continuous cold oxygenated perfusion of DCD hearts has consistently been shown to provide superior preservation and improved functional recovery compared to cold-static storage (157–159). Recently, Choong et al. have demonstrated that hypothermic perfusion supported aerobic metabolism during DCD heart preservation and facilitated superior functional recovery compared to cold-static storage (155). Rosenfeldt et al. subsequently demonstrated that initial reperfusion with a cardioplegic solution designed to minimize IRI followed by hypothermic perfusion could facilitate successful DCD heart transplantation (156). This group has also demonstrated that this technique is effective in the reanimation and preservation of a human DCD human heart (160).

Hypothermic perfusion appears to improve DCD heart preservation compared to cold-static storage; however, a significant limitation of this method is that assessments of organ viability are limited to evaluating the metabolic (lactate metabolism) and biomarker profile of the organ. Since the heart is preserved in a hypothermic and arrested state, assessments of organ function are not possible. Future research is required to determine if hypothermic perfusion provides myocardial preservation equivalent to that of normothermic perfusion, and clarify the role of hypothermic perfusion in DCD heart transplantation.

### Part 3: DCD Heart Evaluation

The traditional approach to cardiac transplantation involves evaluation of heart structure and function within the donor following declaration of brain death (Figure 2A). Similar assessments may be undertaken in the DCD context prior to WLST in order to identify unsuitable organs; however, the heart is subsequently exposed to a profound ischemic insult following donor extubation (24). Consequently, it is necessary to evaluate the DCD heart prior to transplantation in order to identify viable organs.

The DPP approach relies on *ex situ* assessments of organ viability during the preservation interval (Figure 3A). The Transmedics OCS preserves the heart in a non-working mode that prevents assessments of myocardial function to be undertaken; however, this device enables monitoring of oxygen saturation, lactate concentration, aortic pressure, and coronary blood flow. In addition, assessments of coronary anatomy using angiography during ESHP have been reported (161). In the PROCEED II trial, organs were deemed viable and transplanted if the venous lactate level was lower than the arterial level, and the lactate concentration at the completion of ESHP was <5 mmol/L (137). Similar criteria were utilized by Dhital et al. to identify viable DCD hearts for clinical transplantation (22). This protocol is based on previous work that identified an ending lactate concentration <4.96 mmol/L as the best predictor of 30-day graft failure (63% sensitivity and 98% specificity) following DBD heart transplantation (162). These results suggest that a high lactate concentration could accurately identify hearts at risk of posttransplant graft failure; however, a low concentration does not necessarily rule out the possibility of a high-risk heart (139). This is exemplified by a recent case report describing the preservation of a DBD heart on the OCS over an 8.4-h period (144). Despite a normal lactate profile and perfusion parameters, primary graft dysfunction occurred following transplantation that necessitated support with ECMO. Such outcomes emphasize the importance of assessing myocardial function to confirm organ viability before transplantation, particularly when extended criteria or DCD hearts are being evaluated (23, 149). Unfortunately, there are no clinically approved ESHP devices capable of evaluating myocardial function in a physiologic working mode.

Previous research in large animal models has demonstrated the feasibility of utilizing *ex situ* assessments of myocardial function to predict posttransplant graft function (141). Reproducible, reliable, and easily acquired metrics are required to assess myocardial function prior to transplant. Conductance catheters have been used extensively and provide a broad range of myocardial functional parameters that can identify dysfunctional organs with a high sensitivity and specificity (138); however, conductance catheters are expensive, cumbersome, and difficult to utilize clinically. Interventricular balloons have also been used to assess ventricular developed pressure during ESHP (160). The ideal index of contractility should be sensitive to the inotropic state of the heart, but insensitive to loading conditions, heart rate, and heart size (163). Preload-recruitable stroke work (PRSW) is one such parameter that provides a preload-independent assessment of myocardial function (164), and can be measured during ESHP in a physiologic working mode in a non-invasive and automated fashion (165). Two-dimensional echocardiography has also been used to obtain non-invasive assessments of PRSW, fractional area change, and ejection fractions (EFs) as markers of myocardial contraction (142, 166, 167); however, a standardized approach to echocardiography in the *ex situ* perfused heart has not been developed. Overall, such non-invasive approaches could eliminate the need for conductance catheter assessments and facilitate translation of donor heart functional evaluation in the *ex situ* setting to clinical practice. Further advancements in ESHP technology may provide the ability to perform comprehensive functional and metabolic assessments of the donor heart in the future.

The NRP approach to donor heart evaluation involves weaning from veno-arterial ECMO and facilitates assessments of organ function to be carried out within the donor by measuring cardiac output using a pulmonary artery catheter. A comprehensive transesophageal echocardiographic evaluation can also be performed. The NRP approach, therefore, provides an opportunity to directly assess myocardial function and suitability for transplantation following organ resuscitation, rather than relying on metabolic surrogates of organ viability during ESHP. Acceptability criteria for clinical transplantation following NRP include a cardiac index (CI) ≥2.5 L/min/m^2^, central venous pressure ≤12 mmHg, pulmonary capillary wedge pressure (PCWP) ≤12 mmHg, and a left-ventricular EF ≥ 50% on transesophageal echocardiography (23). Interestingly, 3/9 donor hearts that were accepted and transplanted based on these *in vivo* functional criteria exhibited lactate profiles during ESHP that would have deemed the organ non-viable. Further, 1/5 donor hearts that exhibited acceptable lactate profiles required mechanical circulatory support posttransplant (23). These results underscore the value incorporating metrics of myocardial function into pretransplant viability assessment algorithms.

### Part 4: DCD Heart Transplantation

#### Experimental Transplantation

Early investigators sought develop techniques for the resuscitation, preservation, and transplantation of hearts from donors that had suffered an anoxic arrest, in an era when little was known about IRI, immunosuppression, or extracorporeal perfusion (168–171). Christiaan Barnard confirmed the clinical impact of such research, by resuscitating hearts from human DCD donors on cardiopulmonary bypass and subsequently transplanting them (11, 12). However, enthusiasm for research regarding DCD heart resuscitation and transplantation waned over the next 20 years, following the acceptance DBD transplantation (7–9). Heart transplantation soon became limited by a shortage of suitable donor organs and the number of transplants performed annually plateaued (1), which prompted a renewed interest DCD. A number of investigators subsequently published studies describing the successful transplantation of hearts subjected to 17–60 min of warm ischemia following donor exsanguination (70, 172–179). In 2009, however, it was demonstrated that an exsanguination model of donor warm ischemia significantly reduced the severity of myocardial injury sustained by the heart compared to the more clinically relevant model of donor extubation (112). In this context, the clinical translation of studies that employed an exsanguination model of donor warm ischemia is difficult.

Gundry et al. utilized a more clinically relevant model of hypoxemic cardiac arrest in lambs and demonstrated that donor pretreatment with dextrose, methylprednisolone, prostaglandin E_1_, and nifedipine could facilitate successful transplantation following 40 min of warm ischemia (180). They employed the same protocol in baboons and reported posttransplant survival ranging from 1 to 34 days (181). Donor pretreatment with an endothelin-A receptor antagonist and an ATP-sensitive potassium channel opener have also been shown to facilitate successful DCD transplantation in dogs (93, 182). While these studies provided experimental evidence that hearts sustaining significant periods of warm ischemia following donor extubation could be transplanted, donor pretreatment is ethically prohibited in most jurisdictions.

In 2006, Osaki et al. utilized a clinically relevant model of DCD (donor extubation and no pretreatment) and demonstrated that controlled reperfusion with a tepid blood cardioplegia could facilitate successful transplantation in pigs (39). To minimize donor heart exposure to additional ischemia following procurement, the hearts were continuously perfused in a beating state during preservation and implantation. The same group demonstrated that the addition of a ROS scavenger to the initial reperfusion solution minimized lipid peroxidation and improved posttransplant function (98). Iyer et al. reported successful transplantation of DCD pig hearts exposed to 30-min of warm ischemia following donor extubation (140). These hearts were resuscitated with Celsior solution supplemented with erythropoietin, glyceryl-trinitrate, and zoniporide, and then preserved on the Transmedics OCS. We have also demonstrated the importance of a cardioprotective resuscitation strategy and ESHP to minimize incremental ischemic injury during preservation and transplantation (94). Finally, Ali et al. demonstrated that DCD hearts resuscitated using NRP could be successfully transplanted, with outcomes comparable to hearts from DBD donors (183). These translational studies provided evidence to suggest that DCD heart transplantation could be successfully performed when an approach to donor heart resuscitation, preservation, and transplantation is tailored to the DCD context (Figures 2C and 3).

#### Clinical Transplantation

The first report of clinical DCD heart transplantation in the modern era was published by Boucek et al., describing three pediatric transplants performed between 2004 and 2007 at Denver Children’s Hospital (184). Each donor had suffered birth asphyxia and a decision to WLST was made based on the futility of ongoing care. The donor was monitored for progression to cardiorespiratory arrest and mechanical asystole. A 3-min standoff period was observed in the first donor; however, this was shorted to 1.25 min in the subsequent donors based on the recommendations of the ethics committee. The mean time from extubation to declaration of death was 18 ± 8 min. Cold preservation fluid was then infused though a balloon catheter placed in the ascending aorta and venous exsanguination was undertaken. Posttransplant inotropic support, rejection episodes, ventricular function, and 6-month survival were comparable to control infants who underwent DBD heart transplantation during the same period. This report prompted vigorous ethical debate in the transplant community regarding the definition of circulatory death, whether cardiac transplantation from DCD donors violates the “dead donor rule,” and the minimum standoff period duration that must be observed before death can be declared (185–187).

Ali et al. reported on a DCD donor with a 23-min WIT that was resuscitated using normothermic cardiopulmonary bypass, following exclusion of the cerebral circulation (188). After 5 min of reperfusion the heart spontaneously reverted to sinus rhythm. The patient was re-intubated and 190 min later was weaned from cardiopulmonary bypass. Following insertion of a pulmonary artery catheter, a CI of 2.4 L/min/m^2^ was measured with a pulmonary artery capillary wedge pressure of 13 mmHg. The authors suggested that hearts from Maastricht category III donors might be suitable for use in clinical transplantation. This report again prompted ethical debate surrounding the postmortem restoration of mechanical cardiac function using cardiopulmonary bypass in a donor that had been declared dead based on cardiorespiratory criteria (189). However, the technique of NRP has become an accepted means of facilitating multi-organ DCD retrievals in some countries (190).

Iyer et al. utilized the DPP approach to DCD heart resuscitation in a donor that had suffered a 32-min WIT (191). The heart was reperfused with a resuscitative cardioplegia and then transferred to a modified Transmedics OCS device for ESHP and functional assessment in a physiologic working mode. The heart produced a cardiac output of >5 L/min with a left atrial pressure of 14 mmHg. Rosenfeldt et al. also reported on a DCD donor heart that was reperfused with a resuscitative cardioplegia after 32 min of warm ischemia, and preserved using hypothermic ESHP during transport (160). The heart subsequently underwent ESHP and functional evaluation over a 12-h period. Messer et al. utilized the NRP approach in three CD donors with a mean time WIT of 28 min, and demonstrated that *in vivo* measurements of myocardial function correlated with assessments conducted *ex situ* on a modified Transmedics OCS device (192). Finally, Osaki et al. compared the myocardial function of four DCD hearts with five DBD hearts that had been declined for clinical transplantation due to the presence of coronary artery disease, advanced age, and donor social history (193). The DCD hearts sustained a WIT of 34 ± 3 min, were reperfused with a standard cardioplegic solution, and then underwent cold-static storage for 152 ± 55 min before evaluation in an ESHP device. The DBD hearts were arrested with the same cardioplegic solution and underwent cold-static storage for 211 ± 31 min before *ex situ* evaluation. Despite the additional cold ischemic insult sustained by the DCD hearts, the recovery of myocardial function was not significantly different compared to the DBD hearts. These reports provided early clinical evidence that hearts from DCD donors could be successfully resuscitated, and formed a foundation on which clinical transplant programs could be developed.

Dhital et al. reported the first clinical adult DCD heart transplants in the modern era (22). These three transplants represented direct clinical translation of the groups’ research in pharmacologic post-conditioning in large animal models (32, 53, 64, 72, 96, 97, 140). Based on these studies, the authors considered Maastricht category III donors <40 years of age with a WIT <30 min (97). WLST occurred in the intensive care unit, an anesthetic bay, or an adjacent operating room, with standoff periods ranging from 2 to 5 min depending on the jurisdiction of donation within Australia. The WITs ranged from 22 to 28 min, with FWITs between 11 and 21 min (Table 2). Donor hearts were resuscitated using the DPP approach and St. Thomas’ cardioplegia supplemented with erythropoietin and glyceryl-trinitrate (97). Unfortunately, zoniporide is not approved for clinical use and could not be included in the cardioplegia. Donor hearts were preserved on the Transmedics OCS for 245–260 min prior to transplantation. A perfusate lactate concentration <5 mmol/L and evidence of myocardial lactate extraction were used as evidence of myocardial viability. Two recipients required temporary mechanical circulatory support posttransplant; however, all patients were weaned from support and discharged from hospital after 21–28 days. Two patients experienced a rejection episode, but all patients demonstrated normal biventricular function at follow-up (Table 3). A fourth donor failed to progress in the pre-determined time frame and demonstrated a rising lactate concentration during preservation on the OCS; therefore, this heart was not considered for transplantation. This report represented the first clinical evidence that hearts from DCD donors could be resuscitated, preserved using ESHP while being transported from a distant site, and transplanted.

**Table 2:** Donation and pre-transplant characteristics for clinical transplants performed with hearts donated following circulatory death.

Donation and pretransplant characteristics															
	Messer et al. (23)									Dhital et al. (22)			García-Saez et al. (194)		Summary
Age (years)	33	28	38	29	38	43	32	36	44	26	26	27	39	21	33 ± 7
Sex (M/F)	M	M	M	M	M	M	F	M	F	M	M	M	M	F	79% M
BSA (m^2^)	1.88	2.00	2.35	2.38	1.92	2.08	1.80	1.94	1.68	2.14	1.83	2.00	2.22	1.75	2.0 ± 0.2
WIT (min)	60	18	29	17	28	24	21	146	23	28	25	22	14	36	35 ± 34
FWIT (min)	17	12	25	16	24	18	19	16	13	21	20	11	13	21	18 ± 4
NRP Duration (min)	52	52	190	61	34	27	45	29	40	–	–	–	–	–	59 ± 50
*In vivo* CI (L/m^2^)	3.2	2.8	2.9	4.1	3.6	3.5	2.9	4.5	2.8	–	–	–	–	–	3.4 ± 0.6
*In vivo* HR (beats/min)	85	122	92	135	105	125	118	100	148	–	–	–	–	–	114 ± 21
*In vivo* PCWP (mmHg)	12	9	11	8	11	8	10	6	8	–	–	–	–	–	9.2 ± 1.9
*In vivo* EF (%)	58	66	70	60	68	70	59	67	66	–	–	–	–	–	65 ± 5
OCS Duration (min)	170	173	0	170	184	166	139	428	209	257	260	245	360	307	219 ± 104
Initial A lactate (mmol/L)	8.7	2.1	–	5.1	4.4	6.8	3.5	12.1	2.5	8.3	6.8	7.6	5.3	6.2	6.1 ± 2.8
Initial V lactate (mmol/L)	12.5	1.9	–	5.1	5.9	7.3	3.6	12.6	2.9	8.1	6.5	7.4	4.9	6.0	6.5 ± 3.2
Final A lactate (mmol/L)	7.3	1.3	–	4.5	0.2	7.6	4.2	10.3	3.3	3.6	2.8	2.7	2.5	4.0	4.2 ± 2.8
Final V lactate (mmol/L)	7.2	1.2	–	4.4	0.2	6.9	3.4	10.3	3.3	3.6	2.3	2.5	2.5	4.0	4.0 ± 2.7

*BSA, body surface area; CI, cardiac index; EF, ejection fraction; FWIT, functional warm ischemic time; HR, heart rate; NRP, normothermic regional perfusion; OCS, organ care system; PCWP, pulmonary capillary wedge pressure; WIT, warm ischemic time*.

**Table 3:** Recipient and posttransplant characteristics for clinical transplants performed with hearts donated following circulatory death.

Recipient and posttransplant characteristics															
	Messer et al. (23)									Dhital et al. (22)			García-Saez et al. (194)		Summary
Age (years)	59	23	61	58	58	64	55	51	41	57	43	57	52	26	50 ± 13
Sex (M/F)	M	M	M	M	F	M	M	M	F	M	F	M	M	M	M 79%
BSA (m^2^)	2.08	2.03	1.89	2.00	1.73	1.94	1.84	1.98	1.72	1.77	1.86	1.91	1.89	1.66	1.9 ± 0.1
Diagnosis	DCM	HCM	DCM	HCM	DCM	DCM	DCM	DCM	RVC	DCM	DCM	AVRD	DCM	DCM	DCM 71%
VAD	No	No	No	No	No	No	No	Yes	No	No	No	No	Yes	Yes	Yes 21%
TPG (mmHg)	7	4	7	8	8	5	8	8	7	7	5	8	7	8	6.9 ± 1.3
PVR (Woods Units)	1.9	1.3	1.9	2.2	3.0	1.3	2.8	2.1	2.2	1.0	1.7	2.2	1.8	1.5	1.9 ± 0.6
IABP (days)	1	0	0	9	0	0	0	0	0	1	0	2	0	0	1 ± 2
VA ECMO (days)	0	0	0	7	0	0	0	0	0	4	0	0	0	0	1 ± 2
ICU LOS (days)	4	5	4	29	5	5	7	5	4	7	9	6	8	32	9 ± 9
Hospital LOS (days)	20	15	19	80	17	38	17	29	26	26	28	21	62	46	32 ± 19
LV function	N	N	N	40%	N	N	N	N	N	N	N	N	N	N	N 93%
RV function	N	N	N	N	N	N	N	N	N	N	N	N	MOD	MOD	N 86%

*AVRD, arrhythmogenic right ventricular dysplasia; BSA, body surface area; DCM, dilated cardiomyopathy; HCM, hypertrophic cardiomyopathy; IABP, intra-aortic balloon pump; ICU, intensive care unit; LOS, length of stay; LV, left ventricle; MOD, moderate dysfunction; N, normal; PVR, pulmonary vascular resistance; RVC, right ventricular cardiomyopathy; RV, right ventricle; TPG, transpulmonary gradient; VAD, ventricular assist device; VA ECMO, veno-arterial extracorporeal membrane oxygenation*.

**Table 4:** Alternative approaches to the resuscitation of hearts donated after circulatory death.

	Direct procurement and perfusion
Pro	Composition of initial reperfusion cardioplegia can be tailored to minimize ischemic reperfusion injury
	Conditions of initial reperfusion cardioplegia delivery can be tailored to minimize ischemic reperfusion injury
	*Ex vivo* reanimation associated with fewer ethical objections
	Repeated assessments of organ viability can be performed during the preservation interval

Con	*Ex vivo* perfusion required for all procured organs to assess viability prior to transplant
	Unable to assess myocardial function prior to transplant (with currently available technology)

	**Normothermic regional perfusion**

Pro	Expeditious reperfusion
	Ability to assess heart function *in vivo* prior to organ procurement
	*Ex vivo* perfusion only required for organs deemed viable
	May reduce ischemic organ injury and increase the number of usable organs from a donor

Con	Additional equipment and personal required to manage extracorporeal membrane oxygenation circuit
	Exposure to high levels of catecholamines present in donor blood during reperfusion
	Isolation of cerebral circulation required prior to reperfusion
	Ethical objections in some countries/regions

García-Saez et al. described two adult clinical DCD heart transplants into recipients who were bridged to transplant using a durable left-ventricular assist device (194). The authors considered Maastricht category III donors <50 years of age. The WITs ranged from 14 to 36 min, with FWITs between 13 and 21 min (Table 2). Donor hearts were resuscitated using the DPP approach and Custodial cardioplegia supplemented with erythropoietin and glyceryl-trinitrate. Donor hearts were preserved on the Transmedics OCS for 307–360 min prior to transplantation. Neither recipient required mechanical circulatory support postoperatively. At hospital discharge both recipients displayed normal left-ventricular function and moderate right ventricular dysfunction (Table 3). This report demonstrated that hearts from DCD donors could be successfully transplanted into recipients bridged to transplant with an implantable left-ventricular assist device.

Finally, Messer et al. described nine adult clinical DCD heart transplants performed using the NRP approach (23), clinical translation of previous research in large animal models (183). The authors considered Maastricht category III donors <50 years of age; however, donors with WITs extending beyond 30 min were considered. The WITs ranged from 17 to 146 min, with FWITs of 12–25 min (Table 3). Hearts were resuscitated using veno-arterial ECMO and weaned from support after 27–190 min. A CI ≥ 2.5 L/min/m^2^, with a CVP ≤ 12 mmHg, PCWP ≤ 12 mmHg, and an EF ≥ 50% were used as evidence of viability. Donor hearts were then preserved on the Transmedics OCS for 0–428 min prior to transplantation (one local donor heart underwent a brief period of cold-static storage prior to transplant and was not preserved on the OCS). Hearts preserved on the OCS underwent continuous perfusion during transplantation to further minimize exposure to ischemic injury.

Two recipients required mechanical circulatory support posttransplant; however, all patients were weaned from support and discharged from hospital after 15–80 days (Table 3). Interestingly, three of the hearts displayed lactate profiles during preservation on the OCS that suggested non-viability (22, 137); however, these hearts were deemed viable based on *in vivo* functional assessments and transplanted (one heart required intra-aortic balloon pump support for 1 day). Conversely, the heart that required veno-arterial ECMO following transplant displayed an acceptable lactate profile during preservation on the OCS. These results highlight the need for further research regarding assessments of organ viability prior to transplant. Importantly, this study also demonstrates that DCD hearts with extended WITs may still be considered for transplantation, provided the FWIT remains <30 min and acceptable myocardial function is demonstrated. Finally, the authors utilized continuous myocardial perfusion during transplantation. In the PROCEED II trial, donor hearts were exposed to 83 min of additional cold ischemia during implantation (137). Therefore, utilizing continuous perfusion during implantation may significantly improve posttransplant outcomes.

These recent clinical reports have demonstrated encouraging short-term outcomes following DCD heart transplantation; however, the impact of the warm ischemic insult sustained following WLST on long-term outcomes is unknown. Ischemic injury occurring during DBD heart transplantation has been linked to the development of graft vasculopathy and myocardial fibrosis (195–197), and further research is required to determine if similar sequelae will be observed with DCD hearts.

## Conclusion

The recent clinical reports of DCD heart transplantation have demonstrated encouraging short-term outcomes using Maastricht category III donors, with 100% hospital survival following a mean hospital length of stay of 32 days. The limited data currently available suggest that 29% of DCD heart transplant recipients will require temporary mechanical circulatory support; however, all patients have been successfully weaned from support and the majority exhibit normal biventricular function on follow-up echocardiogram. As the experience with DCD heart transplant grows, assessments of organ viability may improve and the need for posttransplant mechanical circulatory support may be reduced. It appears that both the DPP and NRP approaches to organ resuscitation can be used successfully to facilitate DCD heart transplantation; however, further research is required to determine if one approach is superior to the other. While these short-term results are encouraging, the impact of the warm ischemic insult sustained following WLST on long-term outcomes is unknown. Ischemic injury occurring during DBD heart transplantation has been linked to the development of graft vasculopathy and myocardial fibrosis (82–84), and further research is required to determine if similar sequelae will be observed with DCD hearts.

Advances in our understanding of pharmacologic post-conditioning have facilitated the development of controlled reperfusion strategies that can successfully resuscitate the DCD heart. The clinical availability of the Transmedics OCS now allows donor heart preservation in a beating state and a means to limit exposure to additional cold ischemia prior to transplantation. As technology evolves, the ability evaluate DCD hearts during EHP will improve our ability to identify viable organs. Investigating ways to optimize the resuscitation, preservation, evaluation, and long-term outcomes of these hearts is vital to ensure a broader application of DCD heart transplantation in the future.

## Author Contributions

CW, SM, SL, JC, DHK, and DJK: manuscript preparation and revisions. JN and DHF also contributed to manuscript preparation and revisions.

## Conflict of Interest Statement

The authors declare that the research was conducted in the absence of any commercial or financial relationships that could be construed as a potential conflict of interest.

## Abbreviations

ATP, adenosine triphosphate; DBD, donation after brain death; DCD, donation after circulatory death; DPP, direct procurement and perfusion; ECMO, extracorporeal membrane oxygenation; ESHP, *ex situ* heart perfusion; IRI, ischemia-reperfusion injury; MPT, mitochondrial permeability transition; NCX, sodium–calcium exchanger; NHE, sodium–hydrogen exchanger; NRP, normothermic regional perfusion; OCS, organ care system; RISK, reperfusion injury salvage kinase; ROS, reactive oxygen species; PRSW, preload-recruitable stroke work; SAFE, survivor activating factor enhancement; SR, sarcoplasmic reticulum; WLST, withdrawal of life-sustaining therapy.
